# On the Mechanism Determining the Th1/Th2 Phenotype of an Immune Response, and its Pertinence to Strategies for the Prevention, and Treatment, of Certain Infectious Diseases

**DOI:** 10.1111/sji.12175

**Published:** 2014-05-21

**Authors:** P A Bretscher

**Affiliations:** University of SaskatchewanSaskatoon, Saskatchewan, Canada

## Abstract

It is well recognized that the physiological/pathological consequences of an immune response, against a foreign or a self-antigen, are often critically dependent on the class of immunity generated. Here we focus on how antigen interacts with the cells of the immune system to determine whether antigen predominantly generates Th1 or Th2 cells. We refer to this mechanism as the ‘decision criterion’ controlling the Th1/Th2 phenotype of the immune response. A plausible decision criterion should account for the variables of immunization known to affect the Th1/Th2 phenotype of the ensuing immune response. Documented variables include the nature of the antigen, in terms of its degree of foreignness, the dose of antigen and the time after immunization at which the Th1/Th2 phenotype of the immune response is assessed. These are quantitative variables made at the level of the system. In addition, the route of immunization is also critical. I describe a quantitative hypothesis as to the nature of the decision criterion, referred to as the Threshold Hypothesis. This hypothesis accounts for the quantitative variables of immunization known to affect the Th1/Th2 phenotype of the immune response generated. I suggest and illustrate how this is not true of competing, contemporary hypotheses. I outline studies testing predictions of the hypothesis and illustrate its potential utility in designing strategies to prevent or treat medical situations where a predominant Th1 response is required to contain an infection, such as those caused by HIV-1 and by *Mycobacterium tuberculosis*, or to contain cancers.

## Preface

Contemporary frameworks to understand immune class regulation appear to me inconsistent with a series of pertinent observations, made at the level of the system and most described over 40 years ago. Such frameworks would seem justifiable only if these observations are held to be invalid. I believe most of these earlier observations have disappeared from the collective consciousness of immunologists. I therefore describe what I regard as older, under-appreciated but critical observations, in greater detail than I would if I thought them better known. I also believe that the immunological community would better contribute to the relief of human suffering, if it embraced a more integrated, and therefore a more valid perspective than that offered by the contemporary, dominant frameworks. I attempt to illustrate why I believe this. I develop a particular framework and illustrate its potential utility in designing strategies of immunological intervention.

## Preliminary considerations

What is the biological significance of the existence of distinct classes of immunity? Two facts are surely pertinent: different classes are differentially regulated, and both physiological and pathological observations attest to the importance of the class of immunity generated. For example, the small fraction of individuals, infected by HIV, who generate a sustained and exclusive cell-mediated response, do not develop AIDS, whereas the large majority of infected individuals, whose immune response evolves into an antibody, humoral mode, do 1.

An early set of seminal observations came from studying leprosy patients. Their clinical symptoms, and their expression of different classes of immunity, led to a recognition of the importance of the differential expression of cell-mediated immunity, as assessed by DTH reactions to *M. leprae* antigens, and the presence of antibody specific for these antigens. Two extremes were noted. On the one hand, patients with tuberculoid leprosy had low bacterial burdens, expressed strong DTH reactions, and had low levels of antibody. These individuals were not very sick. On the other hand, patients with lepromatous leprosy had high bacterial burdens, expressed weak DTH reactions, and had high levels of antibody. It was recognized that this classification was inadequate, as there were many patients who could not be assigned to these two extreme categories. Other categories of patients were defined, envisaged as existing between these two extremes 2. These early investigators did not look at the IgG subclasses of antibody produced, and only employed DTH skin tests to assess the strength of cell-mediated immunity. Nevertheless, their observations indicated the importance of the class of immunity, generated upon infection, in defining the clinical course of the infection 2.

The underlying simplification of this description is that there are only two classes of immunity, differentially expressed in different patients, and that this differential expression is an important determinant in the clinical course of the infection. We now know that there are in humans seven main classes/subclasses of antibody, IgA, IgE, IgM and IgG_1_–IgG_4_, and that the production of these is differentially regulated. This fact attests to the sophistication of the mechanisms controlling the class/subclass of immunity generated. Nevertheless, the idea that there are two major classes of immunity provided the setting for major advances.

Mosmann's and Coffman's discovery, that clones of murine CD4 T cells can be classified into two subsets, was a major step forward. Cells of these two subsets are distinguished by the cytokines they produce upon stimulation with antigen, and the classes/subclasses of antibodies whose production they support 3,4. The Th1 and Th2 subsets respectively produce the signature cytokines IFN-γ and IL-4. Cells of Th1 clones can mediate DTH on transfer to unprimed mice that are challenged with antigen. Parasite-specific CD4 Th1 cells can deliver IFN-γ to class II MHC-bearing and parasite-infected macrophages, resulting in the macrophages’ activation and killing of the parasites. Cells of the Th1 subset can enhance the production of murine IgG_2a_ antibody under certain circumstances, as described below. The delivery of IL-4 to B cells by Th2 cells can enhance their production of IgG_1_ and IgE antibodies 4. A critical step was observations demonstrating the relevance of these *in vitro* findings to the *in vivo* situation 5. Mice of different strains, infected with the standard number of a million *Leishmania major* parasites, either contain the infection, and are designated as resistant, or suffer chronic/progressive disease, in which case they are designated as susceptible. Containment is associated with expression of DTH to leishmanial antigens, and with parasite-specific CD4 T cells that produce IFN-γ, whereas progressive disease is associated with the predominant production of IgG_1_ antibody and parasite-specific CD4 T cells that produce IL-4 5. Mice infected in a manner that they generate an exclusive ‘Th1’ response, that is their CD4 T cells produce IFN-γ but not detectable IL-4, do not produce detectable IgG_2a_ antibody, but mice that generate a predominant Th1 response predominantly produce IgG_2a_ antibody, and mice that generate a predominant Th2 response predominantly produce IgG1 antibody 6. Thus, the predominant production of IgG_2a_ antibody appears to reflect a predominant Th1 response. Similarly in humans, the existence of DTH without significant production of antibody 2 means that the predominant production of IgG_2_ antibody most probably reflects a predominant rather than an exclusive Th1 response 7.

I shall focus here on how the Th1/Th2 phenotype of an immune response is determined, as there are more pertinent observations at hand on the generation of Th1 and Th2 cells than of other CD4 T cell subsets. However, I would first like to make a few observations on the generation of some other, more recently defined, CD4 T cell subsets, and will also add a few further comments, once I have discussed how the generation of Th1 and Th2 responses might be regulated.

Certain gut flora are required to allow the development of substantial Th17 responses 8. PAMPs expressed by these gut flora are likely involved in facilitating these responses. APC associated with the intestinal tract have special properties, such as their production of retinoic acid; this production is likely significant for the generation of IL-10- and TGFβ-producing T_reg_ cells from naïve CD4 T cells 9. These observations indicate that PAMPs can play a role in immune class determination and illustrate that the route of immunization can be critical due, at least in part, to different types of APC at different immunological sites. The latter conclusion illustrates that the nature of the APC can be important in determining the class of immunity generated.

## Control of the Th1/Th2 phenotype of immune responses

### Immune deviation

Immune deviation was discovered in the 1960s and appears central to understanding immune class regulation. Asherson and Stone first demonstrated that the production of antibody to an antigen precluded subsequent attempts to induce delayed-type hypersensitivity (DTH), a cell-mediated response, to the antigen 10. They invented the term immune deviation. I refer to their phenomenon as humoral immune deviation to indicate, in the name, the direction of the deviation. Somewhat later, Parish demonstrated the converse phenomenon of cell-mediated immune deviation. Rats could be immunized to express DTH to an antigen and become partially unresponsive for the induction of antibody 11, in studies described in greater detail below. The existence of cell-mediated and humoral immune deviation leads to three questions: What is the mechanistic basis of these deviations? What is the mechanism determining whether antigen induces a cell-mediated or humoral response? And what were the evolutionary pressures that resulted both in the existence of these different classes of immunity and in their differential regulation? I formulated a theory 12 in the early 1970s that proposed answers to these questions. I still employ this theory to navigate my understanding of the immune system.

I was greatly influenced by Chris Parish's observations. I landed up in the same department as Chris, in Australia, as an indirect result of our common interests.

## Mechanisms of exclusivity: the cellular basis of states of immune deviation

Ian Ramshaw, Chris Parish and I explored the basis of humoral and of cell-mediated immune deviation in the mid-1970s. We found, in modern terminology, that humorally immune mice harbour CD4 T cells that ‘suppress’, in an antigen-specific manner and on transfer to normal mice, the induction of a cell-mediated response, in the form of DTH. We refer to such cells as TsDTH cells 13,14. We also showed that DTH is mediated by CD4 T cells 14. We thus defined two classes of CD4 T cells that differ in three characteristics: their ability to mediate DTH, their ability to suppress the generation of DTH responses and the conditions under which they are generated. These observations, made with polyclonal CD4 T cells, are presumably related to the subsequent discovery, about a decade later, of Th1 and Th2 clones 3,4.

**Figure 1:**
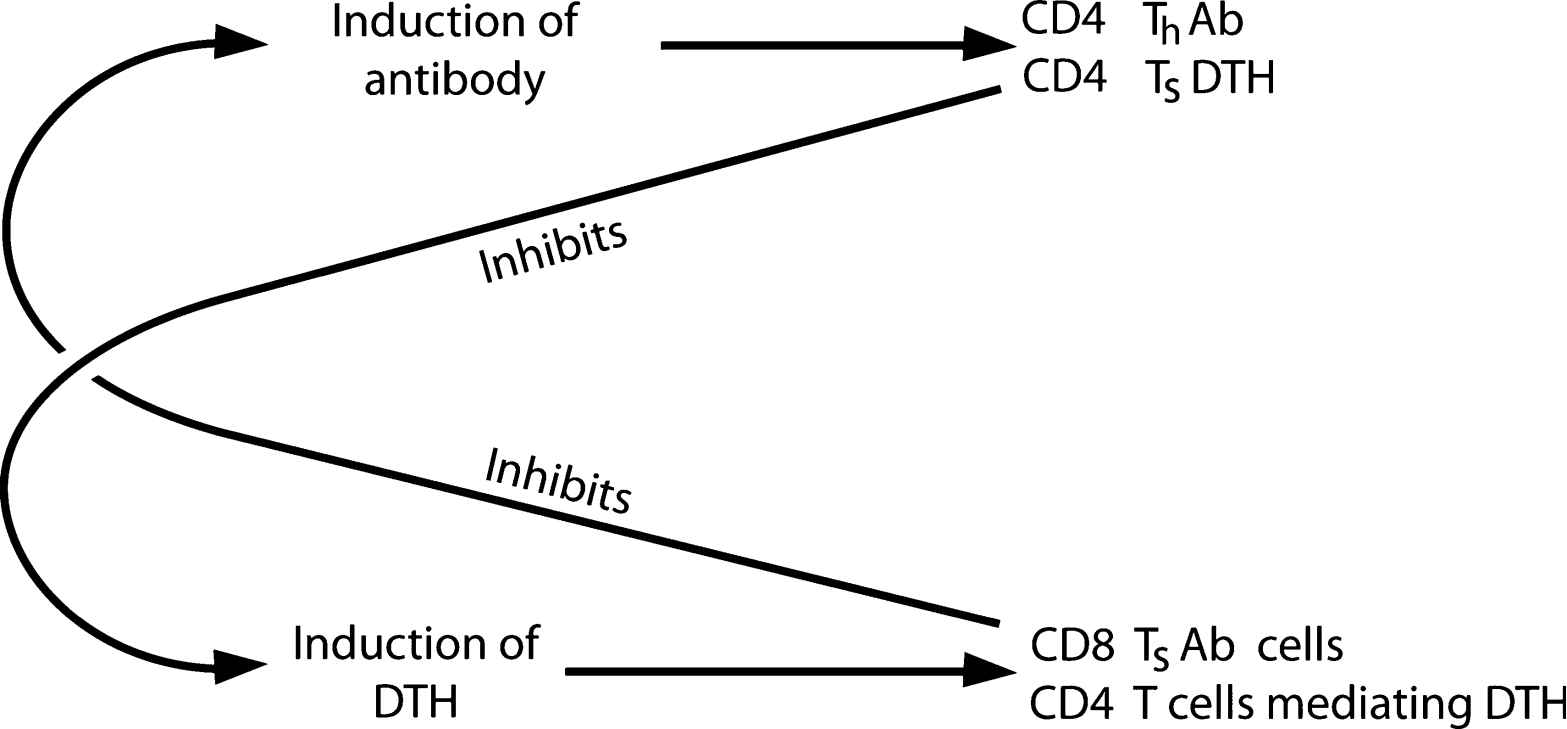
Summary of findings pertinent to the role of TsDTH and TsAb cells in mediating the exclusivity of DTH and antibody responses.

Conversely, mice, in a state of cell-mediated immune deviation, harbour CD8 T cells that ‘suppress’, on transfer to normal mice, the induction of an antibody response 14,15. We refer to such cells as TsAb cells. Later, the existence of ‘suppressor’ CD8 T cells became a focus of the greatest contention 16,17. Nevertheless, I have had no compelling reason, in the intervening years, to doubt the legitimacy of our observations 18. Our findings 13–15 tested proposals put forward in my Theory of Immune Class Regulation 12 and are summarized in Fig.1.

## The ‘decision criterion’ controlling whether antigen induces a cell-mediated or an antibody response

A believable description of how antigen interacts with cells of the immune system, to determine the class of immunity induced, should account for why certain variables of immunization affect the class of immunity generated. I considered in the early 1970s what mechanism might constitute this ‘decision criterion’, by examining what putative mechanisms could account for the variables then known. I describe these variables to set the scene for discussing the hypothesis I proposed.

**Figure 2:**
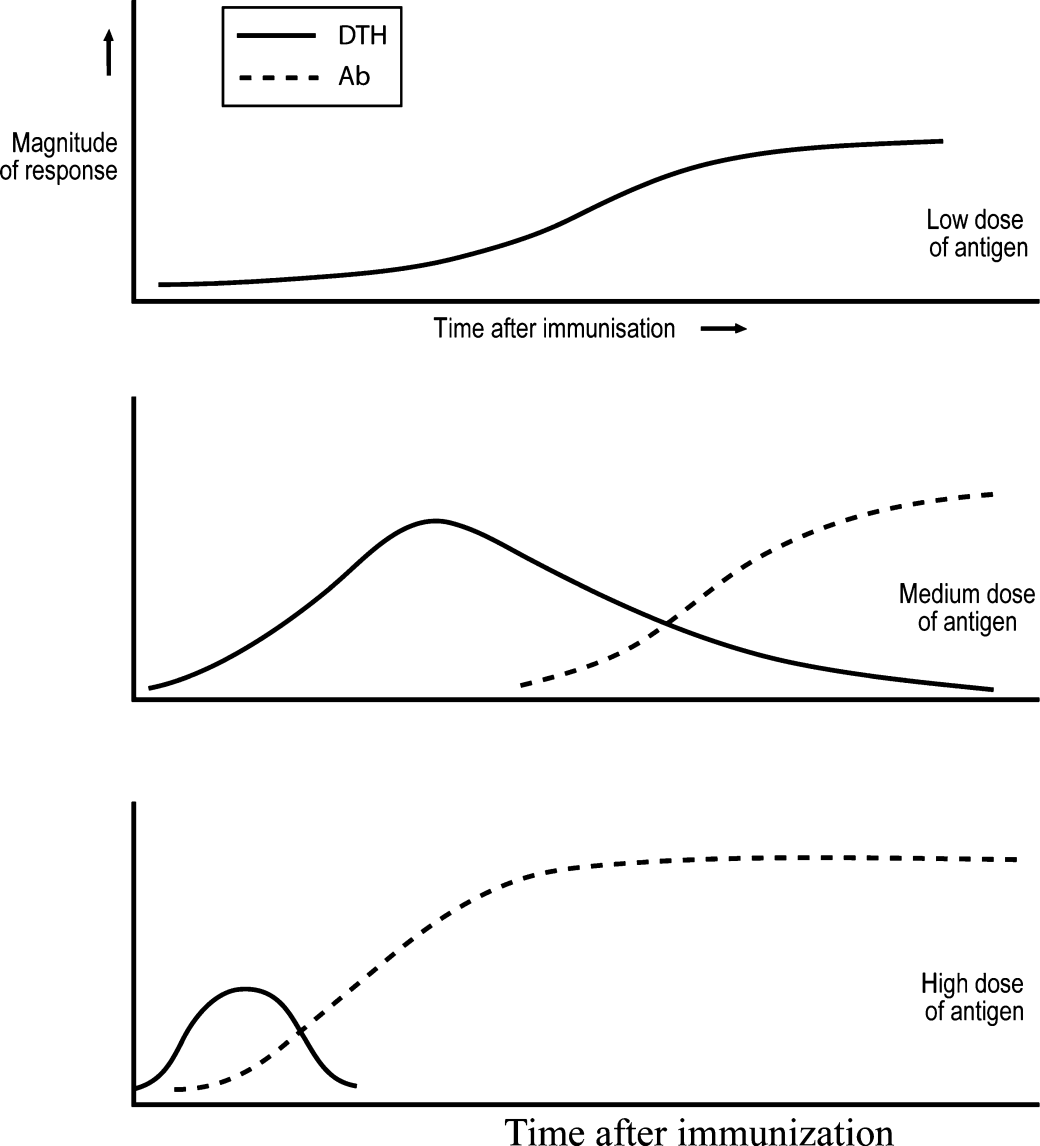
How the DTH and antibody nature of the immune response depends upon antigen dose and time after immunization.

Salvin, in the 1950s, examined how the dose of antigen administered affected the class of immunity expressed at different times following immunization. Figure2 summarizes his conclusions 19. Note that low doses generate an exclusive cell-mediated, DTH response; medium doses more rapidly generate a cell-mediated response that evolves, with time, into a humoral mode; the administration of even larger doses results in more rapid responses, sometimes resulting in a barely detectable cell-mediated phase. These observations reflect what seem to be surprizingly general rules 20. For example, these generalizations appear true for diverse routes of antigen administration, for diverse antigens such as xenogeneic red blood cells 21, the protozoan parasite *Leishmania major* 22, for mycobacteria given to adult 23 or neonatal mice 24, and in different species of animal immunized 25, in the instance referenced, in cows.

**Figure 3:**
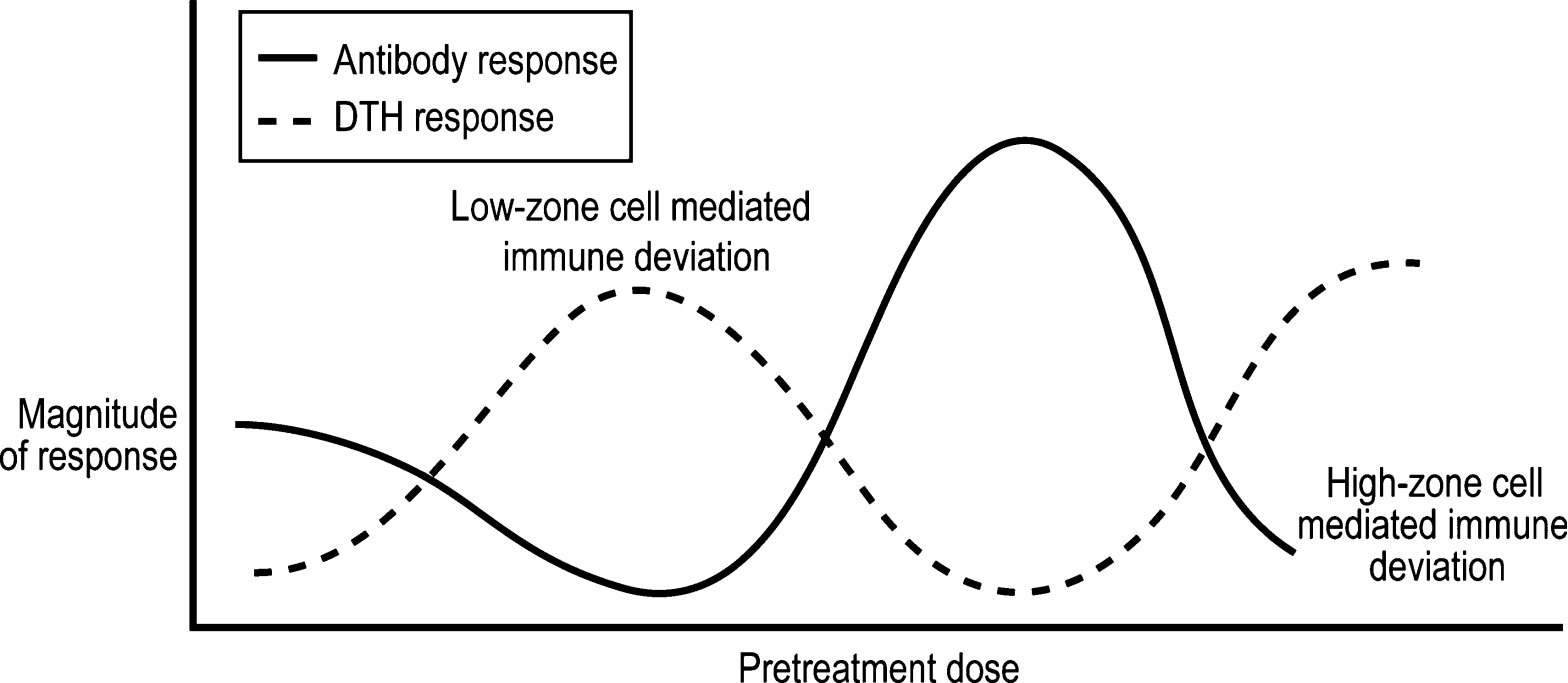
Observations showing the phenomena of low-zone and high-zone cell-mediated immune deviation. For detailed explanation, see text.

These observations can be related to other findings. Mitchison had examined in the mid-1960s how the repeated administration, to immunologically competent mice, of different doses of BSA, affects the nature of the antibody response to a subsequent challenge of BSA that induces an antibody response in naïve mice. Administration over a few weeks of very low or high doses of the antigen inhibited the antibody response to the subsequent challenge, whilst intermediate doses primed the mice for a secondary, antibody response, see the unbroken line of Fig.3. Mitchison, believing the unresponsiveness he observed reflected states corresponding to self-tolerance, dubbed these unresponsiveness states as states of ‘low-zone’ and ‘high-zone paralysis’ 26. Parish carried out similar experiments, but employed a different protein antigen and administered it to rats. He made, however, one additional and crucial measurement. He examined the degree of expression of antigen-specific DTH in his rats at the time of the final challenge. He found that rats exposed to very low and high doses of antigen expressed DTH at the time of the challenge, whereas unexposed rats, or those exposed to intermediate doses of antigen, did not 11, see Fig.3. It seemed to Parish, and to me when I read of his work, that both his and Mitchison's observations, on the generation of unresponsiveness at the level of antibody production, reflected states of low-zone and high-zone cell-mediated immune deviation. Parish's observations were in accord with those of Salvin, in that doses, subimmunogenic for antibody production, induced DTH.

Note that Salvin's observations were made after animals were immunized once with antigen. A ‘lock’ into a cell-mediated immune mode, resulting in cell-mediated immune deviation, only seems to occur after repeated immunizations over an extended period, with very low, or high, doses of these non-living antigens. I would suggest such low-dose antigen exposure reflects what happens following an infection by a very few, non-rapidly replicating, live organisms.

It seems that low amounts of complex antigens, such as SRBC 21, or infection with a low number of slowly replicating organisms, such as mycobacteria 23,24 and *Leishmania major* parasites 22, can induce DTH, Th1 responses, but it is very difficult to generate DTH, Th1 responses by giving larger amounts of these antigens, or infecting with greater numbers of these organisms, than those able to induce antibodies. It may well be that the DTH responses observed by Mitchison and Parrish, on repeatedly administering high doses of antigen, were only attainable because the antigens chosen are relatively non-immunogenic, not being very foreign. I think the induction of DTH/Th1 responses by low amounts of these antigens or lower numbers of organisms is likely to be much more physiologically important than the DTH/Th1 responses generated upon administration of high doses of weakly immunogenic antigens.

These studies, by Mitchison and Parish, did not look at the class of antibody produced. These investigators measured the levels of IgG antibody by its capacity to bind antigen. Few studies of this era looked at the subclass of IgG antibody produced. As already noted, more recent studies employing *Leishmania major* parasites 22 and mycobacteria 23,24 show that infection with low numbers can generate potent and exclusive Th1 responses, without the production of detectable antibody, whereas infection with higher, but still relatively low, numbers generates predominant Th1 responses, with the generation of a few Th2, IL-4-producing CD4 T cells, and predominant production of IgG_2a_ antibodies 22–24.

The importance of these observations, on the different effects of different doses of antigen in generating different types of immunity, lies, in part, in their quantitative nature. If successfully incorporated into a conceptual framework, they would make quantitative considerations central to the field.

Pearson and Raffel made a pertinent generalization, also of a quantitative nature, that was critical to my thinking. They suggested that certain antigens were only able to induce cell-mediated immunity. They identified these antigens as being minimally foreign, due either to their small size or, being larger, being only slightly different from a self-antigen. Moreover, antibodies to such molecules could be raised if these molecules were chemically coupled to a more foreign antigen and animals immunized with the conjugate 27. I was tentatively led to the same proposition that Pearson and Raffel had stated as an experimental generalization, from a more conceptual point of view, as I shall now relate.

## Evolutionary pressures

The Two Signal Model of lymphocyte activation provides a ready explanation for how foreign antigens, that cross-react with a peripheral self-antigen, can sometimes induce autoimmunity against the peripheral self-antigen, see my related article on peripheral tolerance at the level of CD4 T cells 28. One aspect of this situation troubled me. Cells, infected by intracellular pathogens, cross-react with the corresponding uninfected cells and so have the potential for inducing autoimmunity against the uninfected cell. It would seem such situations often arise. The troubling and paradoxical aspect of our considerations can be summarized thus. A driving force, underlying the formulation of the original Two Signal Model of lymphocyte activation, was to understand how peripheral tolerance could be achieved. As might be hoped, such a model, constructed to account for peripheral tolerance, might also account for some of the circumstances that lead to autoimmunity. We have seen that the Two Signal Model did not fail in this respect. However, the circumstances under which autoimmunity might be induced, namely the impingement upon the immune system of a foreign antigen cross-reacting with a peripheral self-antigen, would seem to occur frequently. In this sense, the explanation of peripheral tolerance seemed ‘too leaky’ to be satisfactory, in that autoantibodies would often be induced. Moreover, reports indicated the frequent occurrence of autoantibodies when sensitive techniques are employed for their detection. It was in the context of such conflicting considerations that a finding struck me.

Humphrey and Dourmashin had carefully estimated that several 100,000 IgG antibody molecules had to bind to a red blood cell to allow complement to be activated and so lead to the lysis of the red blood cell 29. This remarkable finding led me to consider three questions. Is this requirement necessary on a purely mechanistic basis, or has this requirement arisen as a result of evolutionary forces? I strongly opted for the latter possibility, as I imagined molecular mechanisms could not be so intrinsically limiting. Secondly, I also realized that this requirement meant IgG autoantibody would be benign unless specific for antigen expressed densely on self-cells. This requirement obviously also limits the usefulness of IgG-dependent, complement-mediated lysis to contain a foreign invader to those situations in which the foreign antigens recognized were densely expressed on cells of the invader. The second question was whether foreign cells, with a sparcity of foreign antigens on their surface, and so minimally foreign, would induce IgG antibody, as the IgG antibody would likely be ineffective? Pearson and Raffel's paper contained the suggestion not only that IgG antibody would *not* be induced by such antigens, but that cell-mediated immunity *would,* a thought I had tentatively entertained. Their suggestions would only make biological sense if minimally foreign cells were susceptible to a cell-mediated attack, a proposition I therefore considered in the form of the third question: are minimally foreign antigens susceptible to cell-mediated, but not to antibody-mediated, attack? Moreover, some observations then prominently reported might provide indirect support for this supposition. For example, there was a strong belief at the time, based upon a considerable body of observations, that the tumours studied, mainly caused by carcinogens or oncogenic retroviruses, were susceptible to cell-mediated immunity and that a predominant humoral response often correlated with tumour progression 30. The idea that cancers may be minimally foreign and therefore in general be susceptible to cell-mediated but not antibody-mediated attack is an idea I shall return to later. A study, subsequent to the publication of the theory of immune class regulation, showed that the susceptibility of different targets, with different amounts of MHC antigens on their surface, to CTL and IgG antibody-dependent, complement-mediated lysis, was in accord with these ideas; targets with low amounts of antigen are only susceptible to CTL 31.

In time, I realized that many features of the differential regulation of cell-mediated and antibody responses could be understood from a ‘teleological’, that is evolutionary, perspective, in the form of two ‘rules’.

The first rule is that the immune system should rapidly mount an effective immune response against a foreign invader. This rule accounts for why minimally foreign antigens induce cell-mediated immunity. The induction of a cell-mediated or a humoral response could in principle be effective against a very foreign antigen. However, given the envisaged, stringent requirements needed to activate IgG effector functions, low amounts of IgG antibody would not be effective, and only low amounts of IgG antibody could be generated soon after infection, before much clonal expansion of B cells had taken place. Thus, cell-mediated immunity would be required in a primary immune response against even a very foreign invader shortly after infection. The price to be paid is that any autoimmunity generated against a set of auto-antigens would, in general, be more damaging than the production of antibody against the same set. Once clonal expansion of B cells has occurred, and sufficient antibody can be made so that the antibody can be effective against a very foreign invader, a switch to a humoral mode occurs; the consequence of this switch is that the humoral autoimmunity generated is generally less damaging than the corresponding cell-mediated autoimmunity would be. These considerations led me to suggest a second rule: the immune system does not generate a more vicious response than necessary, to contain an invader, as this would only increase the damaging consequences of any autoimmunity generated. This rule ‘explains’, at the teleological level, why cell-mediated immunity is suppressed as a strong antibody response is mounted against a very foreign antigen 12. This attempt, to relate the nature of the effector function in a meaningful way to the conditions required to generate the corresponding immune response, is an idea to which I shall return when discussing more recent findings.

The above evolutionary considerations are those I entertained when first formulating this theory of immune class regulation 12. If completely valid, these considerations imply that antibody will not be damaging when it recognizes only a few sites, or one site, on an antigen. This proposition is consistent with studies showing a need for greater than a million hapten molecules on a single chicken red blood cell to render the red cell susceptible to anti-hapten IgG antibody-dependent lysis by non-sensitized lymphocytes, in the antibody-dependent cellular cytotoxicity reaction 32. There are other situations, however, where antibody may be destructive without recognizing many sites on the antigen, such as the IgG autoantibodies specific for the acetylcholine receptor seen in myasthenia gravis. Although there may be such exceptions, there are several instances where a Th1 inflammatory response seems more damaging than a Th2 response. A case of interest, to which I shall refer later, is the autoimmune non-obese diabetic (NOD) mouse. Not all mice become frankly diabetic, which appears to be mediated by β-islet-specific CD4 Th1 and CD8 CTL 33. Interestingly, the immune responses of mice genetically crippled so they are unable to make CD28 are highly biased towards the Th1 mode 34, and NOD mice deficient in CD28 have a much higher incidence of diabetes 35. It often seems to be the case that Th1 autoimmune responses are more pathological that the corresponding Th2 responses.

In addition, as pointed out by a reviewer, very early responses are smaller and so less damaging, even if inflammatory in nature, than later, larger responses would be, providing another reason for a cell-mediated to humoral switch. I think it highly likely that, once the differential regulation of different classes/subclasses of immunity has been achieved, different selective forces might result in different features that can be of advantage to the host.

## The Threshold Hypothesis

The Pearson–Raffel generalization, that minimally foreign antigens can only induce cell-mediated immunity 27, must mean, if valid, that the immune system has a means of ‘measuring’ the foreignness of an antigen. On reflection, it seems that such a ‘measurement’ must rely upon the fact that there are more lymphocytes specific for very foreign antigens than for minimally foreign antigens. As I thought it most plausible that CD4 T cell activation involved CD4 T cell cooperation, it seemed natural to explore the possibility that modest CD4 T cell cooperation leads to Th1, cell-mediated responses, and robust cooperation leads to the generation of Th2, antibody responses. This possibility, moreover, naturally accounts for most of the observations on how the class of immunity generated depends upon the dose of antigen administered and on the time after immunization at which immunity is assessed.

Consider first a situation in which an optimal level of antigen and a number of CD4 T cells exists that results in robust CD4 T cell cooperation. This situation would result in the generation of Th2 cells. As CD4 T cell cooperation is antigen-dependent, lowering the amount of antigen reduces CD4 T cell cooperation and so, with a sufficient decrease, the CD4 T cell collaboration will only be modest, so only Th1 cells are generated.

Consider a situation, occurring shortly after antigen has impinged upon the immune system, where modest CD4 T cell collaboration results in the generation of Th1 cells. One of the earliest events following antigen impact is the clonal expansion of CD4 T cells and so, so long as the antigen level is sufficiently sustained, the strength of CD4 T cell collaboration will increase, resulting in an evolution of the immune response from a cell-mediated to a humoral mode, as delineated by Salvin, see Fig.2.

The most dramatic prediction of the Threshold Hypothesis is that a partial depletion of CD4 T cells can result in a modulation of the response from what would have been a Th2 to a Th1 mode. This prediction has been tested in diverse ways, as briefly outlined in the next section.

I have not explained, in terms of the Threshold Hypothesis, how this hypothesis might explain that high doses of BSA (Mitchison), or the protein used by Chris Parish in his studies, can generate cell-mediated responses. I know of no explanation as simple, and therefore as compelling, as the explanation for why low doses of antigen lead to cell-mediated responses. My suggestion relies on two assumptions I think plausible. The first, elaborated upon later, is that the implementation of the threshold hypothesis occurs during step two of the two-step, two-signal model of CD4 T cell activation. The critical cooperation between CD4 T cells is envisaged to be mediated by a B cell. Secondly, the optimal uptake of antigen by the B cell involves cross-linking of the B cell's Ig receptors. I suggest, in the presence of large amounts of antigen, resulting in concentrations above the binding constant of the receptor, such cross-linking does not occur so efficiently, and so antigen uptake is less efficient than it is in the presence of more moderate amounts of antigen. In this case, less antigen may be *presented* by B cells in the presence of large than of moderate amounts of antigen, and so CD4 T cell cooperation would be less robust, and Th1 cells would be generated.

## Testing the Threshold Hypothesis

We, my colleagues, students and I, have, in past and ongoing studies, tested predictions of the Threshold Hypothesis. These studies constitute to my mind strong support for the hypothesis, and bear on the underlying molecular/cellular mechanisms. I briefly outline these studies here.

**Figure 4:**
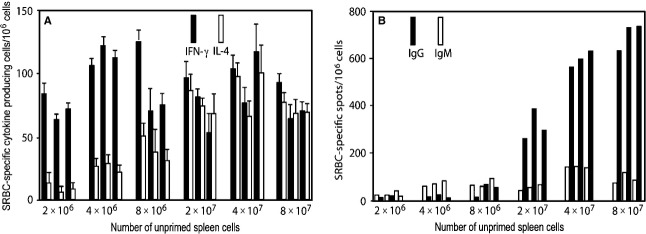
The dependency of the efficiency in the generation of IFN-γ- and IL-4-producing CD4 T cells, panel A, and of IgM- and IgG-producing cells, panel B, on the number of unprimed spleen cells used to reconstitute lethally irradiated mice. All mice were given the same intravenous SRBC challenge. Responses of individual mice, three per group, are shown. Adapted from reference 36.

*In vivo* studies, employing lethally irradiated mice reconstituted with different numbers of naïve spleen cells, but all challenged with the same dose of antigen, given intravenously without adjuvant, demonstrate that a greater number of unprimed spleen cells are required to generate Th2 than Th1 cells 36, see Fig.4. In this figure, the parameter shown, the number of antibody producing cells, or the number of antigen-specific cytokine-producing cells, per million spleen cells employed for reconstitution, reflects the relative efficiency of precursor cell activation (leading to antibody-producing cells or to antigen-specific cytokine-producing CD4 T cells) when irradiated mice are reconstituted with different numbers of spleen cells.

**Figure 5:**
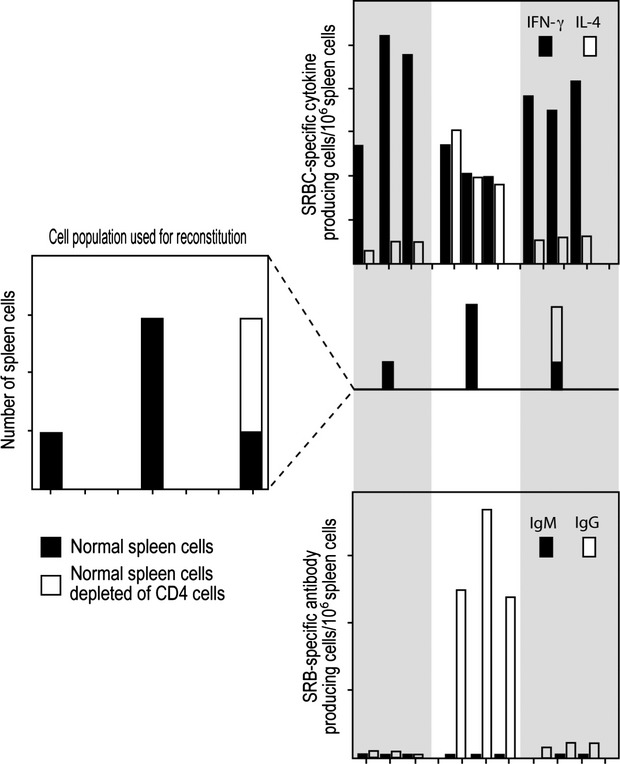
Evidence that the generation of SRBC-specific IL-4-producing Th2 cells requires more CD4 T cells than does the generation of IFN-γ-producing, Th1 cells. The responses of individual mice, three per group, are shown. All mice received the same intravenous challenge of SRBC. Adapted from reference 36. For explanation, see text.

Reconstruction studies show that the cell required in greater numbers, to allow antigen to generate Th2 cells under these circumstances, is a CD4 T cell 36, see Fig.5. In this case, mice are reconstituted with either a small (4 × 10^6^) or a large inoculum (40 × 10^6^) of spleen cells, or with a small inoculum (4 × 10^6^) and additional number of CD4 T cells present in 36 × 10^6^ spleen cells, representing the extra CD4 T cells present in a large inoculum beyond those present in the small inoculum. The observations shown demonstrate that it is the greater presence of CD4 T cells in a large than a small inoculum that is responsible for the more substantial generation of Th2 cells. Moreover, the generation of Th2 cells is dependent in an interdependent fashion on antigen dose and number of CD4 T cells; a lower dose of antigen requires a higher number of CD4 T cells 36. All these observations are anticipated on, and thus support, the threshold hypothesis.

**Figure 6:**
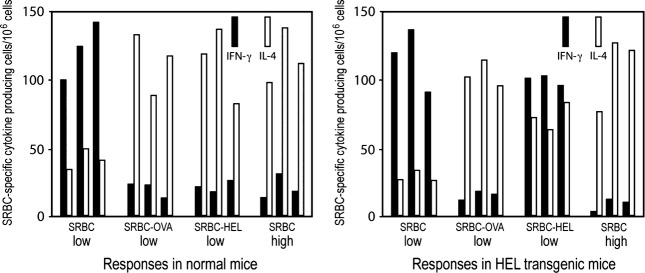
HEL-specific T cells help increase the Th2 component of the anti-SRBC response when intact mice are challenged with HEL-SRBC. Responses of individual mice, three per group, are shown. Adapted from reference 37, For explanation, see text.

Every experimental system has caveats. We have therefore tested the Threshold Hypothesis in diverse experimental systems, including intact mice. Mice given low and high doses of the antigen sheep red blood cells (SRBC), given intravenously without adjuvant, mount, as expected, predominant Th1 and Th2 responses. We attempted to make the antigen ‘more foreign’ by coupling foreign antigens to the SRBC, and determining the effect of this on the Th1/Th2 phenotype of the anti-SRBC response. We coupled ovalbumin (OVA) and hen egg lysozyme (HEL) separately to SRBC, and immunized mice with a *low dose* of each conjugate. In both cases, the conjugates generated Th2 anti-SRBC responses, in contrast to a similar challenge with SRBC that leads to a Th1 response, see Fig.6. We were unsure whether this change in the Th1/Th2 phenotype of the anti-SRBC response was the result of a change in the physical nature of the antigen, due to the conjugating procedure, and/or was the result of recruiting OVA- and HEL-specific CD4 T cells to result in the modulation of the anti-SRBC response from a Th1 to Th2 mode. To sort this out, we also immunized mice that were transgenic for HEL and known to be tolerant towards HEL at the T cell level, with the same antigens. All the anti-SRBC responses were identical in the normal and HEL transgenic mice, except the response on challenge with a low dose of HEL-SRBC. This challenge induced in normal mice a Th2 response and in HEL transgenic mice a more predominant Th1 response 37, see Fig.6. This observation is in accord with the prediction of the Threshold Hypothesis and confirms Pearson and Raffel's proposal that the Th1/Th2 phenotype of the immune response, generated against an antigen, is related to its degree of foreignness.

A number of our *in vitro* observations parallel those made *in vivo*. Different densities of normal spleen cells, cultured with the same dose of antigen, mount different kinds of response. Medium densities allow the optimal generation of T cells that mediate DTH, whereas higher densities allow the same dose of antigen to induce the spleen cells to mount a sustained IgM response with little expression of DTH. Again, the number of T cells appears to be critical in determining the nature of the response 38. These observations, employing polyclonal populations of T cells, parallel the observations made in the adoptive transfer system.

Further and more recent analyses have been carried out *in vitro* with CD4 TcR transgenic cells. It is right to be skeptical concerning the pertinence of *in vitro* observations as a guide to what happens *in vivo*, and to question their physiological relevance. Indeed, the *in vitro* experiments we recently undertook were initiated in part to examine whether, with our own hands, we could repeat some observations reported in a highly quoted study 39. The study showed that low amounts of peptide generate Th2 responses and higher amounts generate Th1 responses. We were skeptical of the physiological relevance of these observations, partly because they were in contradiction with most *in vivo* observations, as illustrated by those of Salvin 19. The culture conditions also seemed rather extreme in that they employed very high numbers of CD4 TcR transgenic cells per culture well. In our studies, we employ an ELISPOT assay 40 that allows us to readily and validly enumerate antigen-specific cytokine-producing cells generated from the activation of a few CD4 T cells. We partially repeated the type of experiment reported in this study 39. We found that, under the conditions employed, about 0.1 antigen-specific, cytokine-producing T cells were produced from each CD4 TcR transgenic T cell plated, whereas CD4 TcR transgenic T cells cultured at lower densities produced a yield of cytokine-producing cells per CD4 cell plated about 100-fold higher 41. The validity of the conclusions drawn from the original study 39 seems questionable, as the *in vitro* conditions employed seem not to have even allowed the clonal expansion of the T cells. We have explored how the Th1/Th2 phenotype of the response is determined when CD4 TcR transgenic cells are cultured under much less dense conditions 42.

The immune response generated in this *in vitro* system shares two features with *in vivo* immune responses. The Th1/Th2 phenotype depends upon the number of CD4 T cells present per culture well, all other variables being held constant, with lower and higher numbers supporting, respectively, the generation of Th1 and Th2 cells. Secondly, keeping the number of CD4 T cells constant, lower doses of peptide support the generation of Th1 cells and higher doses support the generation of Th2 cells. Given these commonalities, we think the *in vitro* system can be used with circumspection to examine the cellular/molecular mechanisms controlling the Th1/Th2 phenotype of the activated CD4 T cells.

Our observations lead us to suggest that B cells mediate the cooperation between CD4 T cells that is critical in determining the Th1/Th2 phenotype of the activated CD4 T cells. The degree of CD4 T cell cooperation is ‘sensed’ via the CD28/B7 system, and not via other candidate costimulator receptor/costimulator systems, such as CD40L/CD40, ICOSL/ICOS and OX40/OX40L 42. The central role we envisage for CD28/B7 interactions is consistent with features already described of CD28 KO mice. These mice have a deficiency of antibody, most of which belongs to the IgG_2a_ subclass, associated with predominant Th1 responses 34.

The presence of IL-4 is required to generate Th2 cells in our system, as in other systems; we show that this IL-4 is produced by the CD4 T cells themselves 42.

## The PAMP and the cytokine milieu hypotheses for how the Th1/Th2 phenotype of activated CD4 T cells is determined

Infection of macrophages by *Listeria monocytogenes* results in the production and secretion of IL-12 by the macrophages. This presence of IL-12 biases the *in vitro* activation of by-stander CD4 T cells towards the production of Th1 cells 43. This production of IL-12 by DC is envisaged to be a direct consequence of infection of the DC 43, or to involve the action of other cells, T cells or innate cells, acting on the DC 44,45. The *in vitro* presence of IL-4 can somewhat similarly bias *in vitro* responses towards the Th2 mode 46. Observations support the idea that IL-4 has a very important *in vivo* role in supporting Th2 responses. The administration of neutralizing anti-IL-4 antibody to mice, infected with *Leishmania major,* can result in a modulation of the response from a Th2 to Th1 mode 47. These kinds of observation have led to substantial explorations of the source of IL-4 required for the generation of Th2 responses 48,49. Observations such as these, in addition to those described above involving *L. monocytogenes,* have led to the idea that the cytokine milieu, in which CD4 T cells are activated, determines their Th1/Th2 phenotype 50. We refer to this possibility as the ‘Cytokine Milieu Hypothesis’.

It is natural, for those who believe that PAMPs/Danger are critical for the activation of CD4 T cells, to consider the potential role of PAMPs/Danger in determining the Th1/Th2 phenotype of the effector CD4 T cells generated. The studies outlined above, showing *L. monocytogenes* can stimulate the production of IL-12 and thereby promote Th1 responses, surely involve some PAMPs of *L. monocytogenes* interacting with host pattern recognition receptors (PRR), that in turn leads to the production of IL-12. We refer to the idea that the PAMPs, associated with an infection, determine the Th1/Th2 phenotype of the effector CD4 T cells generated, as the PAMP hypothesis. The study, employing *L. monocytogenes* and outlined above, illustrates that the PAMP Hypothesis and Cytokine Milieu Hypothesis are not necessarily incompatible 50.

I personally believe that both the PAMP Hypothesis and the Cytokine Milieu Hypothesis are wrong and present an impediment to progress. I want first to take action to try to minimize misunderstandings before I enter this controversial area. I of course do not deny that PAMPs are important in affecting the course and nature of immune responses, or that cytokines have significant roles. Neither do I deny that under some rather unusual circumstances, the cytokine milieu or the presence of PAMPs can be critical. My point of view is that I do not believe that they *are usually the critical factor* as sometimes postulated 50. The belief that they are leads to a neglect of other factors that might be or are important. This neglect could constitute a barrier to constructive exploration.

I summarize below the reasons for my view. A hypothetical mechanism, of how the Th1/Th2 decision criterion operates, should naturally explain the variables of immunization known to affect the class of immunity induced. My concerns are:

1. The immune response against most antigens evolves with time from a cell-mediated, Th1 to a humoral, mixed Th1/Th2 mode. This pattern is seen with pathogens, for example, with HIV 1. As the PAMPs most likely do not change with time after initial infection, the virtually invariable change in Th1/Th2 phenotype of the immune response cannot be reconciled with the idea that the PAMPs, or the intrinsic cytokine milieu, determine the Th1/Th2 phenotype of the response. Moreover, this pattern of how the immune response evolves is also true of foreign, vertebrate antigens, that presumably do not contain PAMPs. The Threshold Hypothesis accounts in a plausible way for this evolution of the Th1/Th2 phenotype of the immune response, and it is natural to suppose the explanation similarly holds for all foreign antigens, independently of whether or not they contain PAMPs.
2. A somewhat similar difficulty appears to exist in trying to understand why the dose of antigen, or the number of non-rapidly replicating organisms employed for infection, similarly determines the Th1/Th2 nature of the immune response. Again, the finding that lower doses or lower numbers induce Th1 responses and higher doses or higher numbers induce Th2 responses seems true of foreign, vertebrate antigens 19,21, that are not expected to contain any PAMPs, and of pathogens, including leishmania parasites 22 and mycobacteria 23,24, that contain a multitude of PAMPS.
3. The idea that pathogens, by virtue of their PAMPS, determine the Th1/Th2 phenotype of the immune response, would, if correct, allow the pathogen to control this phenotype. It would seem in this case that pathogens able to cause chronic infections, by virtue of giving rise to ineffective immune responses, would have an epidemiological advantage over those variants that are well contained. This seems to be a very disadvantageous situation for the host.
4. The most difficult observations to reconcile with these hypotheses are the observations that constitute the most incisive prediction of the Threshold Hypothesis: the diminishment in the number of CD4 T cells, all other variables being held constant, modulates the primary immune response generated from a predominant Th2 to a predominant Th1 mode. Neither the PAMPs, nor the cytokine milieu, are different in these different situations, yet the Th1/Th2 phenotype of the immune response generated is different. Moreover, such modulations occur with foreign, vertebrate, non-PAMP containing antigens, as well as for live *L. major*, as seen in the mouse model 51.

## Coherence

A concept central to strategies aimed at controlling the Th1/Th2 phenotype of the immune response, based upon the Threshold Hypothesis, is ‘coherence’. It is helpful to first illustrate this idea in a well-known context.

The preponderance of different classes of antibody against different epitopes of an antigen, following the induction of an antibody response against the antigen, is usually the same or similar, and in this sense, the class of antibody produced to the different epitopes of an antigen is *coherent*. Fortunately, we can easily understand how this coherence arises. Antigen is endocytosed and presented by different B cells specific for different epitopes of the antigen. The isotype of the antibody, generated by the progeny of all these B cells, is similarly dependent upon the cytokine profile of the CD4 T cells specific for the nominal antigen and involved in the activation of these B cells. Hence, there is coherence in the class of antibody produced against the different epitopes.

Consider what we expect on the Threshold Hypothesis when CD4 T cells, specific for a nominal protein antigen Q, are activated. The strength of CD4 T cell cooperation, for a given Q-specific CD4 T cell, will depend upon the antigen dose and the number of other Q-specific CD4 T cells present. The very same factors will determine the strength of CD4 T cell collaboration for every other CD4 T cell specific for the nominal antigen Q. In other words, the determination of the Th1/Th2 phenotype of the effector CD4 T cells, arising from the activation of all these CD4 T cells, is made on the basis of a *collective* process, rather than processes at the level of *individual* CD4 T cells. Therefore, when Q induces an immune response, it is to be expected that there will be considerable *coherence* in the Th1/Th2 phenotype of the Q-specific effector T cells generated.

The following more extensive considerations are made in response to questions by some reviewers. They are important but more involved considerations than I outlined in the original submission.

Consider now a more complex and realistic situation. The different antigens of a mycobacterium will be present in different amounts, and the number of CD4 T cells specific for these different components will be different. In this case, the same ‘rules’, embedded in the Threshold Hypothesis, applied separately to the CD4 T cells specific for these different antigens, would, if indeed these antigens were not associated with one another, likely result in the generation of effector CD4 T cells with different Th1/Th2 phenotypes. However, this is not what happens according to the Threshold Hypothesis. Consider two mycobacterial proteins, one of which is predominant, P, and one of which is relatively scarce, Q. We assume that P and Q are sometimes associated with one another, as they are parts of the same complex antigen. The P-Q conjugate and P can be taken up by P-specific B cells. Those B cells that take up the conjugate will present both p and q peptides derived from P and Q, whereas B cells that only take up P will present just p peptides. Thus, the activation of a CD4 T cell specific for q will be affected by the presence of the CD4 T cells specific for both p and q peptides, when it interacts with a B cell that has taken up the conjugate P-Q. Thus, the decision as to the Th1/Th2 phenotype of the p- and q-specific CD4 T cells is still a collective, but not a uniform, decision. In this case, the application of the precise rules embedded in the Threshold Hypothesis to the activation of CD4 T cells specific for different nominal components of the mycobacteria, will likely result in some heterogeneity of the Th1/Th2 phenotype of the effector CD4 T cells initially generated. However, our knowledge of the nature of the cytokines produced by different CD4 T cell subsets leads me to suggest that their production ‘sharpens’ up the coherent nature of the immune response. The cytokines produced by a given CD4 T cell subset tend to be ‘self-promoting’ in different ways of this subset: some cytokines promote the generation of cells belonging to the subset producing them, and others inhibit the generation of cells belonging to opposing CD4 T cell subsets. Our observations suggest that IL-4, produced by Th2 cells, is one example of a self-promoting cytokine 42. Thus if 80% of the CD4 T cells initially generated produce IL4 and 20% produce IFN-γ, and these CD4 T cells interact via APC presenting the antigen for which they are specific, I suggest that in time they will give rise to a population of CD4 T cells that more predominantly produces IL-4. The cytokines produced by the majority of the CD4 T cells would self-promote the survival/further generation of their own kind of CD4 T cell.

This type of coherence makes physiological sense. As there are distinct classes of immunity, and these classes are differentially regulated, often with considerable physiological and pathological consequences, it would make little sense if the CD4 T cells specific for the same invader, under given circumstances, are not coherently regulated. The fact of coherence, and the fact that the Th1/Th2 phenotype is determined by ‘collective’ processes, means the Th1/Th2 phenotype of most of the CD4 T cells involved in a response to a given antigen can be easily modulated by changing the antigen dose and/or the number of CD4 T cells.

Finally, a reviewer posed a very good question I would like to respond to: how can what is known about the Th1/Th2 phenotype of responses to allo-MHC fit in with the Threshold Hypothesis? The reviewer pointed out that the frequency of allo-MHC specific CD4 T cells is orders of magnitude greater than for other antigens, and so one would expect, on the Threshold Hypothesis, that allo-MHC antigens would very readily generate Th2 rather than Th1 responses.

Evidence seems to demonstrate a critical role of CD8 T cells in ensuring a Th1 response [52, C. Havele, personal communication]. This fits in with the idea that there is a major role for CD8 T cells in cell-mediated, immune deviation, as discussed earlier, see Fig.1. In addition, in the absence of CD8 T cells, CD4 T cells stimulated by allo-MHC antigen respond in a manner that seems consistent with the Threshold Hypothesis: activation with fewer stimulators favours the more prolonged generation of Th1 cells, but cultures tend to evolve with time towards a Th2 mode. Higher densities of CD4 T cells result in a more rapid transition [C Havele, pers comm.]. It is interesting to note that CD8 T cells, in this role, can also act to achieve coherence for predominant Th1 populations, as they inhibit the generation of Th2 but not Th1 cells.

## Independence

Clinical observations demonstrate that the Th1/Th2 phenotype of the immune response against a pathogen is often critical to whether the pathogen is contained. Given this importance, I thought it likely that the determination of the Th1/Th2 phenotype of the immune responses to two, simultaneous infections, caused by non-cross-reacting pathogens that normally generate different types of response, should be *independently* determined. For example, it would be highly detrimental if, when a nematode infection generates a Th2 response, as usually occurs, this ongoing response readily deviated the primary response upon infection by *M*.* tuberculosis* from a Th1 to Th2 mode. This would result in miliary tuberculosis, lethal unless treated sufficiently early with antibiotics.

We tested this ‘Principle of Independence’. We found conditions where giving one antigen, administered intravenously, generated a Th1 splenic response, and other conditions under which another, non-cross-reacting antigen, also given intravenously, generated a Th2 splenic response. We found that mice injected intravenously with both antigens from the same syringe generated splenic responses indistinguishable from those generated in singly immunized mice. These and other observations support the validity of the principle of independence 53. How can such independence be achieved? It would seem essential that CD4 T cells specific for one antigen do not participate in the decision-making process controlling the Th1/Th2 phenotype of a simultaneous immune response to another, non-cross-reacting antigen. Such specificity in the CD4 T cell interactions would occur if the Th1/Th2 decision-making process obligatorily involves an antigen-specific B cell mediating the critical CD4 T cell cooperation. Such CD4 T cell cooperation may well occur during step two of the envisaged two-step process of CD4 T cell activation.

## Additional comments on the regulation of other CD4 T cell subsets

The ideas outlined above have been deliberately discussed in a simplified context. In particular, we have not taken into full account the existence of distinct classes/subclasses of antibody. I first try to clarify aspects related to the function of Th1 and Th2 cells.

It is often implied in the literature that the exclusive production in mice of IgG_2a_ antibody, and in man of IgG_2_ antibody, reflects an exclusive Th1 response. This is untrue in mice, as very strong DTH, Th1 responses can be generated without the production of detectable antibody 23. The predominant production of IgG_2a_ antibody correlates with a predominant Th1 response, with a measurable Th2 component 6. It also seems likely that in man, an exclusive Th1, DTH response can occur without significant antibody production, as otherwise the exclusive nature of cell-mediated/DTH and antibody responses could not have been so clearly delineated following various infections, such as that caused by *Mycobacterium leprae* 2. It seems likely that predominant Th2 responses correlate in both mice and man with predominant IgG_1_ antibody production, though of course IgE antibody is also produced.

Some of the ideas discussed above, concerning the determination of Th1/Th2 phenotype, might be helpful in understanding how the generation of other CD4 T cell subsets is regulated. I believe my brief remarks here are much more speculative than what I have outlined above concerning how the Th1/Th2 phenotype of an immune response is determined.

1. It seems that Th1 responses can evolve into a mode with a substantial Th2 component, but evolution of the Th1/Th2 phenotype of the response, in the opposite direction, rarely occurs under natural conditions. Evolution of Th phenotype might also be important in understanding the regulation of the allergic response. Those living in a geographical area where allergies to a particular ‘allergen’ are prevalent are either allergic, with predominance of IgE and IgG_1_ anti-allergen antibodies, or non-allergic, with greater predominance of IgA and IgG_4_ specific antibody 54. Desensitization, when it can be realized, involves an evolution of the response from a Th2 to ‘Th3’ mode, with these Th3 cells producing TGF-β and IL-10 54. These cytokines are known to respectively facilitate the production of IgA and IgG_4_ antibody. It appears that when young people grow out of being allergic to milk, there is a shift from a Th2 to Th3-like mode 55.
2. Neither IgA nor IgG_4_ antibody molecules are effective at activating effector mechanisms, such as complement-mediated lysis or mast cell degranulation. Moreover, a study shows that human IgG_4_ molecules are divalent, when first produced, as are all other IgG molecules. However, IgG_4_ molecules are labile in the blood, split into half molecules, and recombine to produce molecules that are monovalent for each of two different antigens. This allows such IgG_4_ molecules, if produced in sufficient amounts, to block the activity of, for example, the IgE-dependent degranulation of mast cells by antigen. Thus, IgA and IgG_4_ molecules not only do not efficiently activate effector mechanisms, but have the potential of blocking the activity of other classes/subclasses of antibody 56. However, it should be noted that IgA can be protective; it protects against gut infections of giardia, an intestinal parasite that causes ‘beaver fever’ 57. I feel it appropriate to refer to an immune state in which Th3 cells predominate, with associated predominant production of IgG_4_ and IgA antibody, as a dominant state of anti-inflammatory immunity. Given these characteristics, it is important that such an anti-inflammatory immune state is not generated acutely after an infection. It seems IgG_4_ production requires chronic stimulation 58. Autoimmune responses tend to be chronic due to a constant source of antigen. It may therefore happen that some autoimmune responses are driven into a minimally damaging, Th3-mode. This may in part explain why deficiencies in natural and inducible T_reg_ cells, with Th3-like activities, are associated with pathological autoimmunity 59. The ideas outlined here may also bear on modern versions of the hygiene hypothesis 60. A less ‘dirty’ environment may reduce Th3 responses and allow more prolonged Th1 and Th2 responses, leading to greater prevalence of Th1 cell-mediated autoimmunity, and Th2 cell-mediated allergies and associated pathological states.
3. A most interesting, longitudinal study, carried out over several years, examined immunity in bee-keepers to bee venom antigens. This study defines a chronic and modulatable, immune state, caused by antigen exposure via non-mucosal routes. Bee-keepers display Th2 immunity against bee venom antigens at the beginning of the bee season. After a few bee stings, antigen-specific IL-10-producing CD4 T cells become activated, leading to the downregulation in these individuals of their Th2 cells. As expected, only IgG_4_, not IgA, bee venom-specific antibody is present, as the responsible regulatory CD4 T cells only produce IL-10 61.

## Strategies of intervention

I very briefly explain, through illustration, why I consider the basic mechanisms, outlined above, may be useful in the rational design of strategies to both prevent and treat various clinical situations.

### The low-dose vaccination strategy against certain infectious diseases

It is well recognized that some infections can only be contained by a cell-mediated, Th1 response, and that an antibody, mixed Th1/Th2 response, results in disease 20. The best-developed mouse model for these human diseases is subcutaneous leishmaniasis, caused by *L. major*. BALB/c mice are considered susceptible, as they rapidly generate, on standard infection with 10^6^ parasites, a predominant Th2 response, associated with progressive disease. We infected BALB/c mice with 10^2^ parasites, resulting in a stable Th1 response and, with time, in a Th1 imprint. Such Th1-imprinted mice make a predominant Th1 response on infection with the standard challenge of 10^6^ parasites, and so resist the infection 6. We thus established a state of low-zone, cell-mediated, Th1 immune deviation, resulting in resistance.

I argue that this strategy should result in effective vaccination against the pathogens causing AIDS 6,20,62 and causing tuberculosis 6,20,63. Mycobacterium-specific Th1 imprints can be readily generated in adult 23 and in very young mice 24.

### The low-dose vaccination strategy against cancer

There are grounds for suggesting the same strategy might be effective against cancer 64,65. A pertinent and well-accepted generalization is that cell-mediated, but not humoral, responses are *usually* effective in protecting against transplantable tumours in experimental animals 64,65.

Studies in the 1950s led to the recognition that animals, given a lethal dose of a syngeneic tumour, often express tumour-specific protective immunity against the tumour some days after implantation 64. This protective immunity is referred to as ‘concomitant immunity’. Robert North, in the 1980s, undertook an extensive analysis of the generation and inhibition of ‘concomitant immunity’. He showed, in a number of different and unrelated tumour systems, that, when mice are given a lethal tumour challenge, concomitant immunity is generated and is apparent about a week or so after tumour implantation. A slowing in tumour growth, or even a temporary decrease in size, often occurs at this time. However, the expression of concomitant immunity is transient. North demonstrated that this was because CD4 T cells arise that suppress concomitant immunity, and so the tumour grows progressively 66. North also made plausible, in the systems he studied, that tumour-specific CD8 CTL were primarily responsible for protection, and no role for antibody could be demonstrated 66. It seemed to me that the first appearance of a protective, cell-mediated response, followed by the appearance and eventual dominance of CD4 T cell suppressor cells, parallels what Salvin had mapped as an initial cell-mediated phase of the response, followed by a humoral phase, see Fig.2. Moreover, we anticipated that, with the evolution of a humoral response, CD4 T cells would arise that suppress the cell-mediated response. We thus entertained the ‘Th2-Skewing Hypothesis of Tumor Escape’. This hypothesis, moreover, explains why the tumours grow progressively, as antibody was believed not to be protective.

We tested critical predictions of this Th2-Skewing Hypothesis in two tumour systems employed by North. There is little doubt that the hypothesis applies in these two tumour systems 67. Establishing a Th1 imprint, by implanting low numbers of tumour cells, results in resistance to a subsequent, normally lethal challenge. There are some observations suggesting the Th2-Skewing Hypothesis might apply in human cancer 64,65, but how general this might be is, to say the least, open to investigation.

### Treatment of infectious diseases caused by pathogens susceptible to Th1 attack

We have seen that the dose of antigen, and number of CD4 T cells, co-determine the Th1/Th2 phenotype of a primary immune response. We have made observations in humans, and carried out experiments in mice, on whether ongoing, mixed Th1/Th2 responses, associated with chronic or progressive illness, can be modulated backwards to a Th1 mode, resulting in a resolution of clinical systems 7,67. Our observations, to be shortly outlined, lead us to suggest that reducing the number of CD4 T cells, and/or lowering the antigen load, can achieve such a modulation. Thus, the number of CD4 T cells and antigen load seem to similarly control the Th1/Th2 phenotype of primary and ongoing immune responses.

Briefly, visceral leishmaniasis, caused by *Leishmania donovani*, is a rapidly fatal disease if untreated. Infected individuals either have a stable, cell-mediated, DTH response, in which case they are healthy, or they become ill, at which time they express little DTH. Both healthy, infected individuals, and patients, have parasite-specific antibodies and so are said to be seropositive. Patients are treated by being administered a course of a highly toxic drug. The drug kills the parasites. Most patients survive the 3-week treatment and, most interestingly, ‘never’ become ill again, even when living in the same endemic area.

We indirectly assessed the Th1/Th2 phenotype of the anti-parasite immune response by examining the relative prevalence of the IgG_1_ and IgG_2_ isotypes among the anti-parasite IgG antibodies. We inferred that healthy, infected individuals had a predominant Th1 response, as expected. Patients, before treatment, had a mixed Th1/Th2 response, also as expected and, after treatment, a predominant Th1 response, indistinguishable from healthy infected individuals. These observations are interesting from three perspectives. They naturally explain why treated individuals do not become ill again. They demonstrate that human immune responses can be modulated ‘backwards’ from a Th1/Th2 to Th1 mode. The success of treatment requires that the parasite be sensitive to the drug, i.e. killed by it. This fact strongly suggests that the modulation of the response from a Th1/Th2 to Th1 mode is a result of a decrease in antigen load 7.

We have seen that infection of ‘susceptible’, BALB/c mice with 10^2^ and with 10^6^* L. major* parasites results, respectively, in a stable Th1 response and rapidly in a highly polarized Th2 response. Partial depletion of CD4 T cells, or the administration of neutralizing anti-IL-4 antibody, close to the time of infection with 10^6^ parasites, modulates the response from a Th2 to Th1 mode, resulting in resistance. Attempts to employ such manoeuvres in mice with established infections, associated with highly polarized Th2 responses, fail to modulate the response from a Th2 to Th1 mode, and so such manoeuvres were considered not to be potential models for treatment of established infections. Most human disease, caused by pathogens whose containment requires a predominant Th1 response, is associated with a mixed Th1/Th2 response, rather than a highly polarized Th2 response. We decided to try and establish, in the mouse model of cutaneous leishmaniasis, a model of chronic disease associated with a relatively stable and mixed Th1/Th2 response, and assess whether, in this case, various treatments, aimed at modulating the immune response, worked. We found that infection of susceptible BALB/c mice with 3 × 10^3^ parasites led, in about 6 weeks, to very large, semi-stable lesions and a semi-stable, mixed Th1/Th2 response. Partial depletion of CD4 T cells at this time, or the administration of neutralizing anti-IL-4 antibody, resulted in a dramatic modulation of the immune response towards a Th1 phenotype, shrinkage of the lesion, and a drop in parasite burden. Recovered mice were highly resistant to a standard challenge with 10^6^ parasites 69. We conclude that ongoing immune responses can be modulated from a mixed Th1/Th2 to a Th1 phenotype by partial depletion of CD4 T cells.

### Treatment of early stages of AIDS

I would like to illustrate why I think these generalizations are of practical importance, by considering how a simple treatment of early stages of AIDS might be realized.

Clinical observations lead me, and some others, to suggest that a predominant Th1, CTL response, is optimally protective against HIV. There are three, direct types of observation supporting this proposition. There are, firstly, seronegative, non-progressors, that have CTL and predominant Th1 responses 68. Secondly, most individuals are not very ill early after infection, and for a time after they seroconvert, a span of time therefore referred to as the honeymoon period. This is a time when the infected individuals have a predominant Th1 response associated with vigorous CTL. Thirdly, there are also seropositive individuals who appear to be non-progressors. These individuals apparently have predominant production of IgG_2_ anti-HIV antibody, indicative of a predominant Th1 response 1,68. All these findings lead me to believe that an ability to modulate the response of early AIDS patients, from a mixed Th1/Th2 to predominant Th1 phenotype, may constitute effective treatment, in a way analogous to that just described for the treatment of visceral leishmaniasis. Giving anti-retroviral drugs lowers the viral load and should, I suggest, initially modulate the immune response towards a Th1 mode. How long this takes could be assessed in each individual by longitudinally assessing when their IgG_2_ antibodies become predominant over their IgG_1_ antibodies among anti-HIV IgG antibodies. It may well be that, once this state has been achieved, further anti-retroviral treatment would undermine the protective, Th1, CTL response. In the case of visceral leishmaniasis, drug treatment lasts only about 3 weeks. Because of the drug's toxicity, the art of medicine has led to as short a period of treatment as possible that is effective. It would be very interesting to examine whether standard anti-retroviral treatment causes a change in the IgG isotype composition, among the IgG anti-HIV antibodies, in the manner I suggest, and whether cessation of anti-retroviral treatment, when IgG_2_ antibody is predominant, does not result in viral rebound, indicating immune control of viral replication.
